# An Improved Generative Adversarial Network-Based and U-Shaped Transformer Method for Glass Curtain Crack Deblurring Using UAVs

**DOI:** 10.3390/s24237713

**Published:** 2024-12-02

**Authors:** Jiaxi Huang, Guixiong Liu

**Affiliations:** School of Mechanical and Automotive Engineering, South China University of Technology, Guangzhou 510640, China; 202010100386@mail.scut.edu.cn

**Keywords:** glass curtain crack deblurring, unmanned aerial vehicle, generative adversarial networks, U-shaped Transformer

## Abstract

Drones have emerged as a critical tool for the detection of high-altitude glass curtain cracks. However, their utility is often compromised by vibrations and other environmental factors that can induce motion blur, compromising image quality and the accuracy of crack detection. This paper presents a novel GAN-based and enhanced U-shaped Transformer network, named GlassCurtainCrackDeblurNet, designed specifically for the deblurring of drone-captured images of glass curtain cracks. To optimize the performance of our proposed method for this application, we have meticulously created the GlassCurtainCrackDeblur Dataset. Our method demonstrates superior qualitative and quantitative outcomes when compared to other established deblurring techniques on both the GoPro Dataset and the GlassCurtainCrackDeblur Dataset.

## 1. Introduction

The proliferation of high-rise structures and skyscrapers has led to the widespread adoption of glass curtain wall systems across the globe. However, the long-term exposure of these structures to high-altitude conditions can give rise to significant safety concerns. To mitigate these risks, it is essential to conduct regular inspections and maintenance of glass curtain walls. Traditional inspection methods, which involve technicians rappelling down the facade with the aid of ropes in search of defects, are not only time-consuming and subjective but also perilous. Among the various defects, cracks in glass curtain walls are particularly significant as they can indicate underlying structural issues.

The use of unmanned aerial vehicles (UAVs) equipped with cameras to document glass curtain wall cracks through photographs or video footage offers a safer and more efficient alternative. Advanced techniques such as edge detection and instance segmentation have been successfully applied to enhance crack detection accuracy. These methods, however, heavily depend on the quality of the images or videos captured by the UAVs.

In practice, UAV operations are often compromised by external factors like crosswinds, turbulence, and operator inexperience, leading to camera vibrations that severely degrade image quality. This degradation, characterized by motion blur, can significantly reduce the effectiveness of glass curtain wall crack detection. Consequently, developing techniques to mitigate image blurring resulting from UAV motion is imperative to ensure the reliability of inspection results.

To address the issue of motion blur in UAV-captured images of glass curtain wall cracks, researchers have explored both hardware-based and software-based solutions. Hardware-based methods often involve adding additional weight to the UAV, which can hinder its maneuverability and effectiveness for the intended inspection tasks. Given the drawbacks of hardware solutions, this paper concentrates on software-based approaches that aim to enhance image quality post-capture.

Kejriwal et al. [1] introduced a UAV video stabilization algorithm that integrates optical flow, Kalman filtering, and a low-pass filter to reduce image blur. Zhan et al. [2] leveraged the power of convolutional neural networks (CNNs) within the realm of artificial intelligence to deblur video footage. Liu et al. [3] employed generative adversarial networks (GANs) as a dedicated deblurring module for high-speed drone imagery, which successfully mitigated camera vibration-induced blur and improved the accuracy of vehicle detection. Sun et al. [4] combined the strengths of GANs with the Transformer architecture, achieving a significant boost in deblurring performance, and the experimental drone studies demonstrated that effective image deblurring greatly enhances subsequent object-tracking capabilities.

### 1.1. Image Deblurring

The generalized expression formulation of the non-uniform blur model is shown by Formula (1):

(1) $$I_{B}=k\times I_{S}+N$$

where $I_{B}$ is a blur image, $I_{S}$ is the sharp latent image, and N is an additive noise. k is a large sparse matrix containing a local blur kernel for each pixel, and × denotes the convolution.

The problem of image deblurring can be categorized into blind deblurring and non-blind deblurring. Initially, research efforts were predominantly focused on non-blind deblurring approaches. Non-blind deblurring operates under the assumption that the blur kernel is known, and it aims to reconstruct the sharp latent image using algorithms such as the Richardson–Lucy method, Wiener filtering, or Tikhonov regularization. While these methods are straightforward and can be effective, they are prone to introducing ringing artifacts when the blur kernel is not precisely estimated [5]. Xu et al. [6] proposed that camera shake is the primary cause of blur, which they modeled as spatially variant. They simplified the model to represent 3D camera motion and leveraged the Fourier transform to expedite the deblurring process. Their experimental results indicated that this approach could successfully remove various types of blurs. Schmidt et al. [7] introduced a cascaded discriminative model for image deblurring, with each stage of the cascade incorporating a Gaussian CRF based on regression tree fields. Their method demonstrated high efficiency and produced state-of-the-art restoration quality at the time. Riegler et al. [8] developed conditional regression models that integrated convolutional neural networks and random forests, enabling the efficient utilization of additional kernel information during training and inference and requiring only a single model to be trained. Their method was shown to handle cases where the blur kernel varies between images effectively. Das et al. [9] conducted a comparative study of the Richardson–Lucy algorithm, algebraic deconvolution, and Basis Pursuit deconvolution, finding that the Basis Pursuit method generally yielded the best results. Rangaswamy [10] introduced Residual Whiteness Measures to address individual white noise disturbances in digital device images. Despite the widespread application of manually defined models in non-blind deblurring, their limitations have constrained the quality of image restoration.

Early blind deblurring methods predicted both the blur kernel and the sharp latent image since the blur kernel was unknown [11]. Predicting the blur kernel for each pixel point is an ill-posed problem, and most previous related studies have used methods that make assumptions about the source of blur, employed image statistics, and utilized heuristics. These methods assume that the motion blur produced by the camera is uniform over the entire image. Zhang et al. [12] introduced a blind image deblurring method based on sparse representation, which effectively regularized the ill-posed inverse problem by leveraging the inherent sparsity of natural images. This method successfully mitigated the unwanted ringing effects that are often a byproduct of traditional deblurring techniques, as demonstrated by their experimental results. Liu et al. [13] conducted an analysis of the changes in the image spectrum due to blurring and, after retrieving the blur kernel, established a convex kernel regularizer. When combined with blind image deblurring deconvolution techniques, this convex kernel regularizer significantly improved deblurring outcomes, as evidenced by their experimental validation. Leclaire et al. [14] compared various sharpness metrics, including global phase coherence and different versions of sharpness metrics, highlighting their impact on blurring, noise, and deconvolution artifacts through experimental evaluation. Pan et al. [15] proposed a blind image deblurring method that exploited the dark channel prior, which is particularly effective in scenarios involving natural, facial, textual, and low-light images. By enforcing sparsity on the dark channel, the method achieved state-of-the-art results and demonstrated versatility across different image types. Yan et al. [16] built upon the dark channel prior by introducing a bright channel prior. By utilizing both the dark-channel and bright-channel priors, their method could efficiently restore images by harnessing information from both light and dark regions. Their experimental results showed that this dual-prior approach was more robust than using the dark channel prior alone.

Recently, the field of blind deblurring has been revolutionized by the integration of deep learning techniques, such as generative adversarial networks (GANs), U-Net, and Transformer architectures. These methods have elevated image deblurring to new heights by harnessing the power of artificial intelligence.

### 1.2. GAN

The concept of generative adversarial networks (GANs) was proposed by Goodfellow et al. [17] in 2014. GANs consist of a generator and a discriminator. Through using the game theory between the generator and the discriminator, they can generate realistic high-resolution images. GANs have thus become the most widely used paradigm for generative modeling. Nowadays, they are also widely used in the field of image deblurring. Kupyn et al. [18] pioneered the use of GANs for motion deblurring with the introduction of DeblurGAN. This end-to-end learning method is based on conditional GANs and content loss. DeblurGAN demonstrated a state-of-the-art performance in terms of structural similarity measurement and visual appearance, while also being five times faster than its competitors when integrated with object detection tasks. Based on DeblurGAN, Kupyn et al. [19] further enhanced the model by incorporating feature pyramid networks, creating DeblurGANv2. This version achieved even better deblurring effects with a complex backbone network and could perform real-time video deblurring with a lightweight backbone network. Ramakrishnan et al. [20] proposed a GAN architecture that features global skip connections and dense architectures, bypassing the blur kernel estimation process and significantly reducing testing time. Chen et al. [21] took a different approach by feeding noisy images to the GAN network for training, using the trained network for noisy sample generation to improve deblurring performance. Zhang et al. [22] applied 3D convolutions in the generator of a GAN network to capture both spatial and temporal information from neighboring frames, thereby improving video deblurring performance. Another innovative approach was introduced by Zhang et al. [23], who combined two GAN models: BGAN, which learns to blur sharp images, and DBGAN, which learns to deblur the images blurred by BGAN. This method achieved an excellent performance on the GOPRO Dataset [24], showcasing the power of GANs in blind deblurring tasks. Liu et al. [25] proposed a GAN architecture with a localized skip connection. The localized skip connection was used to address the motion blurring of concrete crack images by UAVs in accordance with specific characteristics of crack images. Lee et al. [26] applied a similar GAN-based method to solve issues with motion blur in bridge inspection using UAVs. To solve the image deblurring problem in a low-light condition, Sharif et al. [27] combined a dense-attention block and a contextual gating mechanism in a feature pyramid structure with GAN-based architecture to leverage content awareness.

These studies highlight the versatility and effectiveness of GANs in the context of image deblurring, demonstrating their potential to push the boundaries of what is possible in restoring clarity to blurred images.

### 1.3. U-Net

U-Net, introduced by Ronneberger et al. [28] in 2015, has become a benchmark in the field of medical image segmentation due to its innovative encoder–decoder architecture, which resembles the letter “U”. The network’s ability to preserve spatial information and context has made it a popular choice for various image restoration tasks [29], including deblurring. Mao et al. [30] adapted the U-Net architecture for image restoration by employing a deep fully convolutional encoder–decoder structure, achieving impressive results in experimental settings. Liu et al. [31] enhanced the U-Net architecture by incorporating wavelet transforms, which reduced the size of feature maps in the contracting path, striking a balance between the receptive field size and computational efficiency. Chen et al. [32] proposed the use of half-instance normalization in the encoder part of U-Net and connected two U-Nets to improve the network’s image restoration capabilities. Wang et al. [33] fused the strengths of U-Net with the Transformer architecture, enabling the network to capture more global information than traditional convolutional operations, which led to the recovery of more detailed images. Chen et al. [34] proposed NAFNet, which achieves an excellent performance at half the computational cost by simplifying the nonlinear activation function in U-Net. Zhang et al. [35] employed a GAN with U-Net as the generator, which performed exceptionally in deblurring pavement crack images and improved segmentation accuracy.

The integration of U-Net with other deep learning components and the adaptation of its structure for specific applications highlight the network’s versatility and its pivotal role in advancing the field of image deblurring.

### 1.4. Transformer

The Transformer architecture, initially proposed by Vaswani et al. [36] in 2017, has had a transformative impact on the field of natural language processing (NLP) due to its self-attention mechanism [37], which is particularly adept at capturing long-range dependencies [38,39]. This architecture has since been adapted and extended into the field of computer vision, with notable contributions such as the Vision Transformer (ViT) by Dosovitskiy et al. [40] and the Swin Transformer by Liu et al. [41], both of which have laid the groundwork for advancements in image deblurring. Wang et al. [33] introduced the Uformer, a network that combines U-Net with Transformer, and adds a local enhancement window similar to the one used in the Swin Transformer. This modification significantly reduces the computational complexity of high-resolution feature maps while retaining its ability to capture long-range dependencies and enhancing the network’s capability to recover fine details in images. Liang et al. [42] enhanced the Swin Transformer block by incorporating residual connections as a deep feature extraction module. This addition allows the network to summarize features across different levels, resulting in a more refined and detailed recovery of images. Zamir et al. [43] addressed the issue of the quadratic growth in the computational complexity of the Transformer with increasing spatial resolution by introducing a gating mechanism and depth-wise convolution. This innovation effectively reduces the time required for model deblurring, making it a practical solution for real-world applications.

The integration of Transformer-based architectures into the field of image deblurring has opened up new avenues for research and development, leveraging the power of self-attention to capture complex spatial relationships and improve the quality of image deblurring.

This study introduces GlassCurtainCrackDeblurNet, a novel approach that combines Generative Adversarial Networks (GANs) with an enhanced U-shaped Transformer architecture, specifically tailored for the deblurring of drone-captured images of glass curtain wall cracks. To optimize the performance of our proposed method for this specialized application, we have specifically established the GlassCurtainCrackDeblur Dataset.

Through rigorous comparative experiments against other established deblurring techniques on both the GoPro Dataset and the GlassCurtainCrackDeblur Dataset, our method has demonstrated superior qualitative and quantitative outcomes. These results affirm the efficacy of GlassCurtainCrackDeblurNet in addressing the challenge of motion blur in drone imagery, thereby contributing to the precision of glass curtain wall crack detection and the safety and reliability of high-altitude inspections.

## 2. Methodology

### 2.1. GlassCurtainCrackDeblurNet

**Figure 1:**
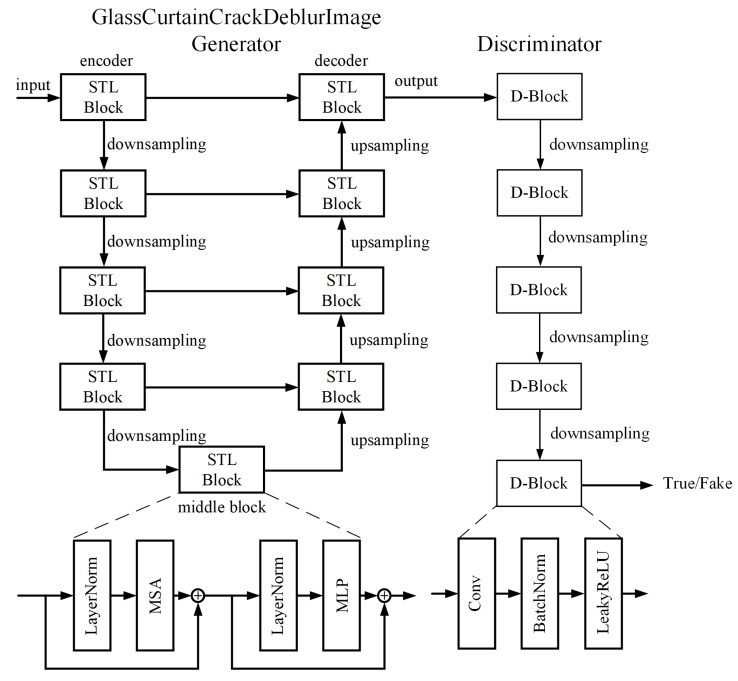
Schematic architecture of GlassCurtainCrackDeblurNet.

To address the challenge of deblurring drone images of glass curtain wall cracks, we propose GlassCurtainCrackDeblurNet, which utilizes GANs as the primary network structure, as demonstrated in Figure 1.

This structure comprises two main components: a GlassCurtainCrackDeblurImage generator and a discriminator. The interplay between the generator and the discriminator, grounded in game theory, enables GlassCurtainCrackDeblurNet to generate realistic, high-resolution images. In our model, the generator takes blurry UAV images as input and generates deblurred images as output. Simultaneously, the discriminator receives both clear UAV images and the generated samples, tasked with discerning between the two. The generator’s objective is to produce deblurred images that are indistinguishable from the true clear images, thereby attempting to deceive the discriminator. The game between the generator G and the discriminator D can be represented by the Formula (2):

(2) $$\underset{G}{\min}\;\underset{D}{\max}E_{x\sim p_{r}}[\log(D(x))]+E_{x\sim p_{g}}[\log(G(x))]$$

wherein $p_{r}$ represents the sampling distribution of the actual UAV images, and $p_{g}$ represents the sampling distribution of the deblurred UAV images. The network structure of the generator G is elaborated in the subsequent sections. The discriminator D adopts the same architecture as in [44] and uses modules in the form of Convolution-BatchNorm-ReLU [45]. Let $C_{k}$ represent a Convolution-BatchNorm-ReLU layer with k filters. Then, the network structure of the discriminator D used in this paper can be represented as: $C_{64}-C_{128}-C_{256}-C_{512}-C_{512}$, with a filter size of 4 × 4. After the last layer, a convolution is applied to map the one-dimensional output, followed by a Sigmoid function.

### 2.2. GlassCurtainCrackDeblurImage Generator

In the GlassCurtainCrackDeblurImage generator G of our paper, we employ an encoder–decoder network with residual connections, commonly referred to as U-Net. This network architecture is designed to effectively extract features from blurred images captured by drones in the encoder part and utilize these features to reconstruct clear images in the decoder part.

**Figure 2:**
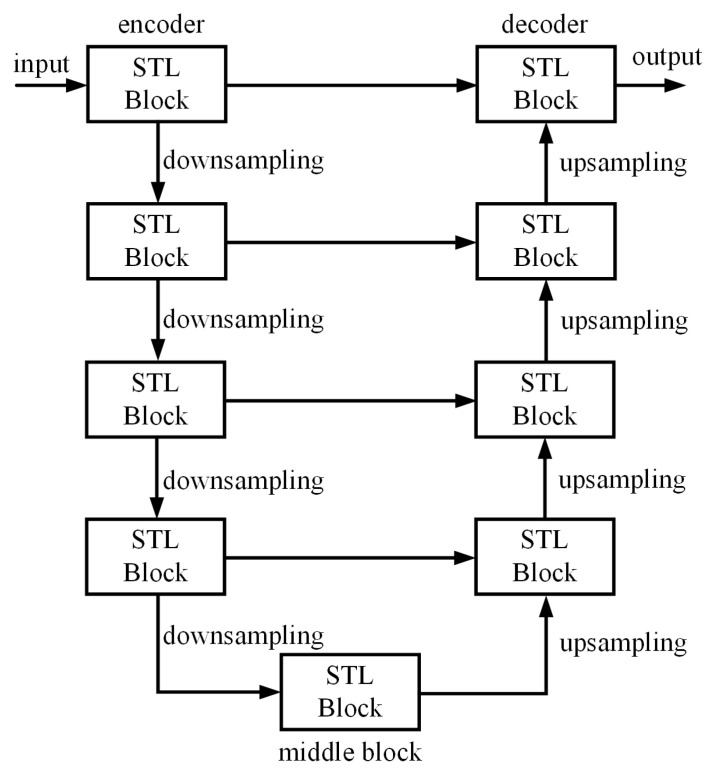
Flow scheme of the GlassCurtainCrackDeblurImage generator G.

As depicted in Figure 2, our GlassCurtainCrackDeblurNet architecture is structured in a U-shaped design [33, 46,47], leveraging the benefits of hierarchical feature extraction and reconstruction. The blurred drone images are initially processed through a 3 × 3 convolutional layer to generate a low-level feature map. This map then undergoes a series of four encoder layers, each composed of a Swin Transformer Layer block and downsampling convolutional layers. The downsampling operation effectively halves the spatial resolution of the feature maps while doubling the number of channels, resulting in feature maps with 64, 128, 256, and 512 channels, respectively, at the outputs of the encoder layers.

To ensure a comprehensive representation of the image’s features, a middle block layer is added at the end of the encoder sequence, which also contains a Swin Transformer Layer block. This hierarchical structure enables the network to capture a wide range of image characteristics.

Following the encoder layers, the feature maps proceed through four decoder layers, each comprising upsampling convolutional layers and Swin Transformer Layer blocks. Prior to being input into the Swin Transformer block in the decoder, the feature maps are concatenated with their corresponding features from the encoder through skip connections. This skip connection mechanism facilitates the fusion of low-level details with high-level semantic information, enhancing the network’s ability to reconstruct the sharp image.

After the decoder layers, the feature maps are further processed through a final convolutional layer, which restores them to clear, deblurred images. The combination of the Swin Transformer Layer blocks and the U-Net architecture allows GlassCurtainCrackDeblurNet to effectively capture both local and global contextual information, leading to improved deblurring performance for drone-captured images of glass curtain wall cracks.

### 2.3. Swin Transformer Layer Block

**Figure 3:**
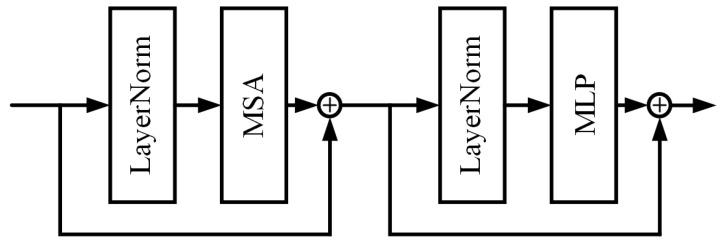
Flow scheme of the GlassCurtainCrackDeblurImage generator G.

Figure 3 illustrates the internal structure of the Swin Transformer Layer (STL) block.

The Swin Transformer Layer block [41] is based on the multi-head self-attention mechanism of the original Transformer block [36], but the main difference lies in the local attention and the shifted window mechanism. Assuming the input feature map size is H×W×C, the Swin Transformer Layer block first divides the feature map into M×M non-overlapping local windows and reshapes it into a feature map of the size $\frac{HW}{M^{2}}\times M\times C$. The number of local windows is $\frac{HW}{M^{2}}$. Then, self-attention operations, or local attention, are performed on each window separately. For a local window feature $X\in {\mathbb{R}}^{M^{2}\times C}$, the calculation of the corresponding matrices Q, K, and V for *query*, *key*, and *value* is as seen in Formula (3):

(3) $$Q=XP_{Q},K=XP_{K},V=XP_{V}$$

Among these, $P_{Q}$, $P_{K}$, and $P_{V}$ are projection matrices shared among different windows. Generally speaking, $Q,K,V\in {\mathbb{R}}^{M^{2}\times d}$. Hence, the attention matrix for the local window is computed through the self-attention mechanism, as seen in Formula (4):

(4) $$\mathrm{Attention}(Q,K,V)=\mathrm{SoftMax}(QK^{T}/d+B)V$$

In this context, *B* represents learnable relative positional encoding. In practical operations, the attention function is duplicated in parallel *h* times and the results are concatenated, serving as the operation for multi-head self-attention (MSA). According to the operation in reference [36], we choose *h* to be 8.

Next, we will employ a multi-layer perceptron (MLP) for further feature transformation. This MLP consists of two fully connected layers and one GELU non-linear transformation layer. A LayerNorm (LN) layer is added before both the MSA and the MLP, and residual connections are added to both modules. The entire process is described as Formula (5):

(5) $$\begin{array}{l} X=\mathrm{MSA}(\mathrm{LN}(X))+X,\\ X=\mathrm{MLP}(\mathrm{LN}(X))+X. \end{array}$$

However, when the partitions of different layers are fixed and do not change, there is a lack of connection between local windows. This limitation can hinder the network’s ability to capture and utilize information across different spatial regions of the image. To address this issue and introduce cross-window connections, we alternatively use regular and shifted window partitions [41]. The shifted window partition involves shifting the features by $\left(\frac{M}{2},\frac{M}{2}\right)$ pixels before performing the segmentation.

## 3. Experiments

### 3.1. Datasets

Given the absence of a dedicated dataset for glass curtain crack deblurring, we created an original dataset, GlassCurtainCrackDeblur Dataset, to facilitate the development of an effective deblurring method. This dataset consists of 2757 pairs of clear and blurred glass curtain wall images for training and 1384 pairs for testing. The creation of this dataset involved the following steps.

**Data Collection**: High-frame-rate clear videos of the Osmanthus Building and CNA Kate Apartments in Shenzhen were captured using the DJI Mavic 2 Pro drone (DJI, Shenzhen, China).

**Image Generation**: Every 5 frames from the videos were combined to create blurred images. This method of blurring simulates the motion blur that drones might encounter during real-world operations.

**Manual Screening**: The resulting images were manually reviewed to ensure the quality and relevance of the data. This process resulted in the inclusion of 4141 pairs of clear and blurred glass curtain wall images in the dataset. The dataset encompasses various types of blurred images, including those with cracks, multi-directional blur, and others, as shown in Table 1.

**Data Augmentation**: Prior to training, data augmentation techniques such as random flipping were applied to the training data. This step enhances the robustness and generalization of the model by introducing variations in the training data and also helps to prevent overfitting.

**Figure 4:**
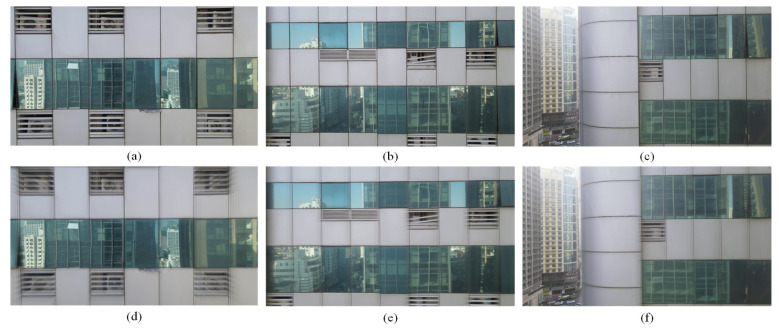
Sample images of GlassCurtainCrackDeblur Dataset: (**a**) one of the sharp images in the dataset; (**b**) one of the sharp images in the dataset; (**c**) one of the sharp images in the dataset; (**d**) one of the blur images in the dataset; (**e**) one of the blur images in the dataset; (**f**) one of the blur images in the dataset.

Figure 4 provides a visual representation of a subset of the GlassCurtainCrackDeblur Dataset, showcasing the diversity and quality of the images used in the training and testing phases of our deblurring method. The creation of this dataset was a critical step in the development of GlassCurtainCrackDeblurNet, as it provides a rich and representative dataset for training and evaluating the performance of the proposed method.

### 3.2. Evaluation Metrics

To evaluate the performance of the model, we used three evaluation metrics: MAE, PSNR, and SSIM. MAE (Mean Absolute Error) is a measure of the average magnitude of the errors in a set of predictions, without considering their direction. It is calculated as the average of the absolute differences between the predicted values and the actual values. In this paper, MAE is used to quantify the difference between the deblurred image and the original image. A lower MAE value indicates a closer match between the deblurred and original images, thus signifying a better deblurring performance. The specific calculation method of MAE is shown by Formula (6):

(6) $$MAE=\frac{1}{mn}\sum_{i=1}^{m}\sum_{i=1}^{n}\left|R(i,j)-G(i,j)\right|$$

where *m* and *n* are the dimensions of the image, and *i* and *j* are the horizontal and vertical coordinates of a specific pixel. *R* represents the clear image, and *G* represents the deblurred image generated by the model.

PSNR (Peak Signal-to-Noise Ratio) is used to evaluate the distortion value of the deblurred image. A higher PSNR value indicates that the deblurred image is closer to the original clear image, with less distortion. The specific calculation method of PSNR is shown by Formula (7):

(7) $$PSNR=10log_{10}\frac{{\left(2^{n}-1\right)}^{2}}{MSE}$$

wherein, as the images used in this paper are RGB images, *n* = 8. The specific calculation method of MSE (Mean Squared Error) is shown by Formula (8):

(8) $$MSE=\frac{1}{mn}{\sum_{i=1}^{m}\sum_{i=1}^{n}\left(R(i,j)-G(i,j)\right)}^{2}$$

The advantage of using PSNR as an evaluation metric is its computational simplicity and its ability to provide a rapid assessment of the deblurred image’s quality. PSNR is based on the error between corresponding pixel values, which makes it a straightforward and efficient way to measure the distortion in an image. However, PSNR has limitations in that it does not directly correlate with human visual perception. This is because PSNR does not account for the structural information in an image, which is often more relevant to the human visual system (HVS).

To address this limitation, this paper also employs SSIM (Structural SIMilarity) as an additional evaluation metric. SSIM is designed to measure the similarity between a deblurred image and the original image in a way that is more aligned with human visual perception. The specific calculation method of SSIM is shown by Formula (9):

(9) $$SSIM=\frac{\left(2\mu_{R}\mu_{G}+c_{1})(\sigma_{RG}+c_{2}\right)}{\left(\mu_{R}^{2}+\mu_{G}^{2}+c_{1})(\sigma_{R}^{2}+\sigma_{G}^{2}+c_{2}\right)}$$

In the formula, $\mu_{R}$ represents the mean value of *R*, $\mu_{G}$ represents the mean value of *G*, $\sigma_{RG}$ represents the covariance between the *R* and *G*, $\sigma_{R}^{2}$ represents the variance of *R*, $\sigma_{G}^{2}$ represents the variance of *G*, and $c_{1}$ and $c_{2}$ are constants used to maintain stability. SSIM ranges from 0 to 1, with higher values indicating a higher degree of similarity between the two images. SSIM takes into account the structure of the image, contrast, and luminance, providing a more perceptually relevant measure of image quality than PSNR.

### 3.3. Training Details

The network in our paper was built on the Pytorch [48] framework v. 1.10.0, working with Python v.3.9.5, and Cudatoolkit [49] 11.3.1. A 32 AMD Ryzen 9 5950X 16-Core Processor (32 GB memory), equipped with an NVIDIA GeForce RTX 3090Ti GPU (24 GB memory), was used in this study. The codes were run on a Windows 10 platform. We used the Adam optimizer [50] to train the model, with $\beta_{1}$ = 0.9 and $\beta_{2}$ = 0.9. The same initial learning rate of 1 × 10^−4^ was used for both the generator and the discriminator, and a cosine annealing schedule [51] was employed to gradually reduce it to 1 × 10^−7^. The training patch size was 256 × 256, with a batch size of 4, and the entire training process lasted for 400 k iterations.

## 4. Results and Discussion

### 4.1. Results of Comparative Experiments

To provide a more intuitive understanding of the performance of GlassCurtainCrackDeblurNet, we conducted both qualitative and quantitative comparisons with state-of-the-art methods that are well regarded in the field of image deblurring: DeblurGANv2, Method [35], and Restormer. These methods were chosen due to their significant contributions and high citation counts in the deblurring domain. The datasets used for comparison were the GoPro Dataset and the GlassCurtainCrackDeblur Dataset proposed in this paper.

The GoPro Dataset provided by [24] was created by capturing 240 fps videos using a GoPro4 Hero Black camera. Blurred images were generated by averaging consecutive frames ranging from 7 to 13, with the middle frame used as the corresponding sharp image. This process resulted in a total of 3214 pairs of blurred and sharp images with a resolution of 1280 × 720 pixels. Out of these, 2103 pairs were used for training, and 1111 pairs were reserved for testing.

#### 4.1.1. Qualitative Evaluation

**Figure 5:**
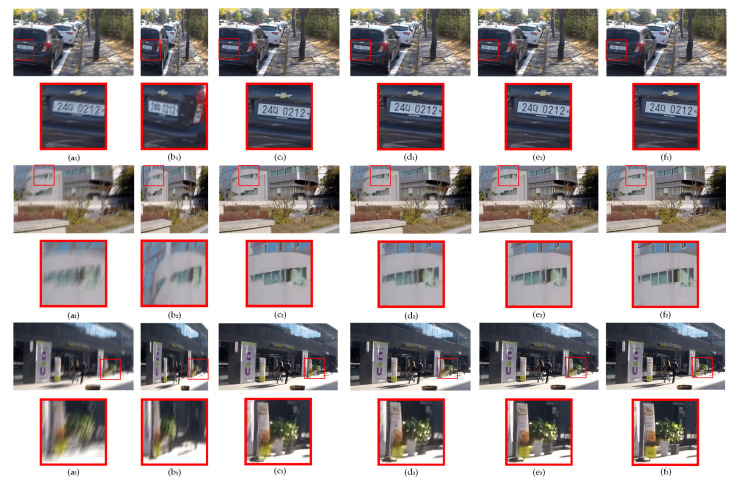
Deblurring results on the GoPro Dataset. The first column (**a_1_**–**a_3_**) shows the input blurred images; the second column (**b_1_**–**b_3_**) shows the output images from DeblurGANv2 [19]; the third column (**c_1_**–**c_3_**) shows the output images from Method [35]; the fourth column (**d_1_**–**d_3_**) shows the output images from Restormer [43]; the fifth column (**e_1_**–**e_3_**) shows the output images from GlassCurtainCrackDeblurNet; the sixth column(**f_1_**–**f_3_**) shows the real sharp images.

The qualitative evaluation results for GlassCurtainCrackDeblurNet on the GoPro Dataset are presented in Figure 5. The red boxes within the images denote areas that have been zoomed in on for closer inspection of the deblurring results.

In the first image, which involves restoring a vehicle’s license plate number, the proposed method demonstrated a superior performance in terms of artifact reduction compared to Method [35] and Restormer. The edges of the license plate numbers restored by GlassCurtainCrackDeblurNet were distinct and sharp, closely resembling the original sharp image. In contrast, the deblurred result from DeblurGANv2 retained more blur, making the license plate numbers less legible.

In the second image, the deblurring result produced by GlassCurtainCrackDeblurNet yielded an overall cleaner image with well-defined and coherent window edges. This demonstrates the method’s ability to maintain sharp boundaries and structure in the deblurred output.

In the third image, GlassCurtainCrackDeblurNet effectively restored green leaves that were both in shape and detail closer to the sharp image. The light spots on the leaves were accurately preserved, while Method’s [35] and Restormer’s deblurring tended to elongate the light spots in the direction of the motion blur, resulting in a less realistic appearance.

These qualitative comparisons illustrate the advantage of GlassCurtainCrackDeblurNet in terms of preserving fine details and reducing artifacts, even when compared to other state-of-the-art deblurring methods.

**Figure 6:**
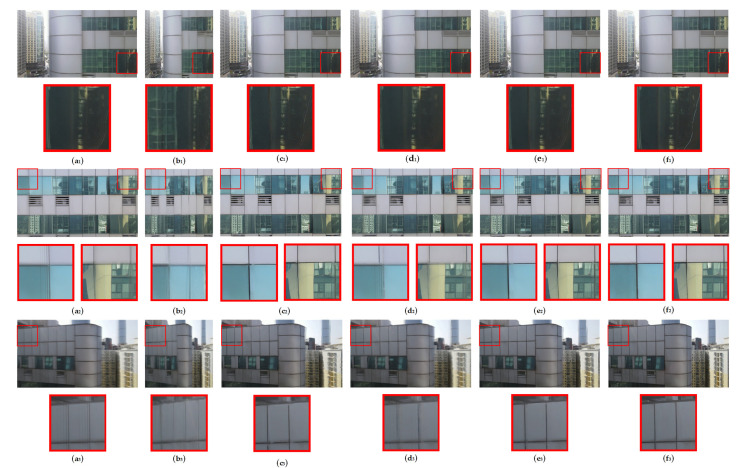
Deblurring results on the GlassCurtainCrackDeblur Dataset. The first column (**a_1_**–**a_3_**) shows the input blurred images; the second column (**b_1_**–**b_3_**) shows the output images from DeblurGANv2 [19]; the third column (**c_1_**–**c_3_**) shows the output images from Method [35]; the fourth column (**d_1_**–**d_3_**) shows the output images from Restormer [43]; the fifth column (**e_1_**–**e_3_**) shows the output images from GlassCurtainCrackDeblurNet; the sixth column (**f_1_**–**f_3_**) shows the real sharp images.

The qualitative evaluation results for GlassCurtainCrackDeblurNet on our proposed GlassCurtainCrackDeblur Dataset are depicted in Figure 6. These results further highlight the method’s effectiveness in handling specific challenges related to glass curtain wall crack deblurring.

For the first image, the other methods, DeblurGANv2 and Restormer, struggled to preserve the target crack during the deblurring process, making it almost indistinguishable. In contrast, GlassCurtainCrackDeblurNet successfully retained the crack, which is sharper than Method [35], demonstrating its capability to preserve important details even in complex scenarios.

In the second image, which featured a complex environment with two directions of motion blur, DeblurGANv2 failed to adequately handle the situation, resulting in a less clear glass frame. Restormer, while attempting to deblur, blurred the glass frame, compromising its clarity. However, GlassCurtainCrackDeblurNet was able to restore a clear glass frame, which was more complete than that restored by Method [35], showcasing its robustness in handling complex environmental conditions.

In the third image, the complexity arose from the increasing blur as the distance between the drone and the glass curtain wall increased. Once again, compared to the other methods, only GlassCurtainCrackDeblurNet was able to restore a clear glass boundary that closely resembled the visual appearance of the sharp image. This capability is particularly valuable for high-altitude glass curtain wall crack detection, where the blur is often more pronounced.

These qualitative comparisons underscore the superior performance of GlassCurtainCrackDeblurNet in preserving crucial details and maintaining clarity in the deblurred images, even in challenging conditions. The visual assessments confirm the purposed method’s ability to achieve a high level of deblurring quality, which is crucial for applications such as glass curtain wall crack detection using drones.

**Figure 7:**
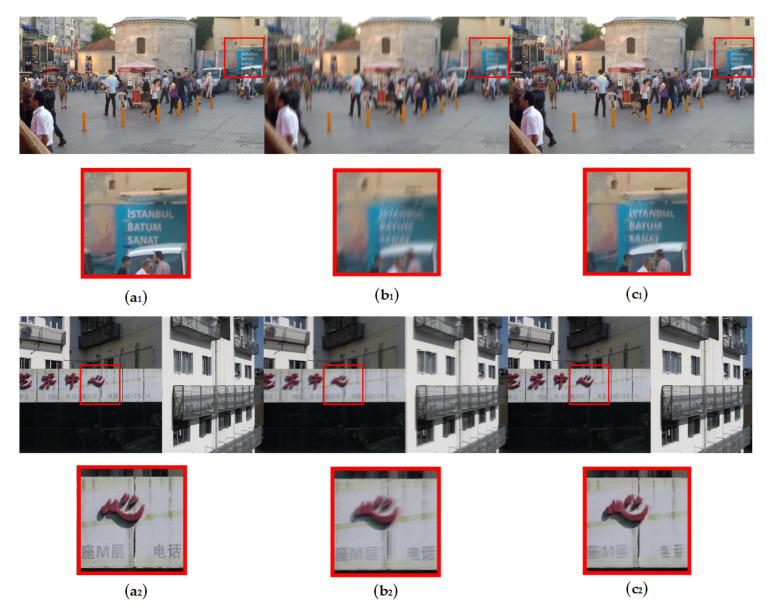
Failure deblurring results for the GoPro Dataset and the GlassCurtainCrackDeblur Dataset from GlassCurtainCrackDeblurNet. The first column (**a_1_**,**a_2_**) shows the real sharp images; the second column (**b_1_**,**b_2_**) shows the input blurred images; the third column (**c_1_**,**c_2_**) shows the failure output images from GlassCurtainCrackDeblurNet.

Additionally, in the deblurring results of the proposed model, we also found some inferior outcomes, as shown in Figure 7. It can be observed that in the two displayed images, the deblurring effect on the text is not good. This might be due to the text having multi-directional edges, which, when combined with motion blur, drastically increases the difficulty of deblurring. Glass curtain cracks, however, rarely have this issue, so this kind of failure generally does not affect the crack detection after deblurring.

#### 4.1.2. Quantitative Evaluation

The quantitative evaluation results for GlassCurtainCrackDeblurNet’s performance on the GoPro Dataset are summarized in Table 2. Our proposed method achieved a PSNR of 29.76 dB, an MAE of 0.0198, and an SSIM of 0.089. These scores place our method at the top of the comparative analysis, indicating a superior deblurring performance. The method with the next best performance, Restormer, achieved a PSNR of 28.94 dB, an MAE of 0.0215, and an SSIM of 0.088.

Additionally, we calculated the average deblurring time for each method. DeblurGANv2 had the shortest deblurring time of 0.12 s, but its deblurring effects were suboptimal. Restormer required 1.97 s, Method [35] took 1.46 s, and GlassCurtainCrackDeblurNet needed 0.93 s. This indicates that our proposed method can achieve better deblurring results with only 47.2% of the deblurring time required by Restormer.

The quantitative evaluation results for GlassCurtainCrackDeblurNet’s performance on the GlassCurtainCrackDeblur Dataset are summarized in Table 3. Our method achieved an MAE of 0.0179, a PSNR of 29.49, and an SSIM of 0.89. These results, once again, demonstrate the superior performance of our proposed method when compared to the other methods tested on the GlassCurtainCrackDeblur Dataset. The method with the next best performance, which is not explicitly mentioned, would be Restormer or DeblurGANv2, depending on the specific metrics used.

Additionally, the average deblurring time for each method for the GlassCurtainCrackDeblur Dataset is provided. Although GlassCurtainCrackDeblurNet did not achieve the shortest deblurring time, it significantly narrowed the gap with DeblurGANv2, which was had the fastest performance on the GoPro Dataset. This indicates that our method has achieved a balance between performance and efficiency, which is crucial for practical applications where both accuracy and processing speed are important.

The quantitative results corroborate the qualitative findings, confirming that GlassCurtainCrackDeblurNet outperforms other state-of-the-art methods in terms of both visual quality and computational efficiency. This efficiency is particularly important for real-time applications, such as drone-based glass curtain wall crack detection, where rapid image processing is necessary.

### 4.2. Discussion

From the comparative results presented, it is evident that while our proposed method, GlassCurtainCrackDeblurNet, may not be as computationally efficient as DeblurGANv2, it excels in terms of deblurring performance. The key to this performance lies in the network’s architecture, which is different from that of GAN-based networks like DeblurGANv2. Our method utilizes an encoder–decoder network with residual connections in the generator. This architecture ensures that after the downsampling feature extraction in the encoder, the decoder performs upsampling feature recovery. This process allows for the generation of a more refined deblurred image of glass curtain crack, as the decoder can utilize the high-level features from the encoder to reconstruct the fine details in the image. A more refined deblurred image with less distortion may improve the PSNR value.

When compared to Restormer, GlassCurtainCrackDeblurNet significantly reduces the time consumption while also offering a superior performance. Our method’s ability to achieve superior results in a shorter timeframe compared to Method [35] and Restormer is attributed to its use of the Swin Transformer Layer (STL) as the basic module for feature extraction. The STL’s self-attention mechanism facilitates the exchange of information between different local features, enabling it to extract both local and global features effectively. With sufficient local and global features, the structural information in a blur image will be considered by GlassCurtainCrackDeblurNet, which may result in an increase in the SSIM value. The utilization of the STL in our method not only enhances feature extraction but also simplifies the network structure, leading to a reduction in deblurring time. This optimization is a significant advantage, as it allows for faster processing while maintaining or even improving the deblurring performance.

In summary, while GlassCurtainCrackDeblurNet may not be the fastest method in terms of processing speed, its performance in deblurring glass curtain crack images is superior to that of DeblurGANv2, Method [35], and Restormer. Table 4 compares the purposed method with other approaches. The integration of GANs and U-Net architecture with the Swin Transformer Layer enables our method to achieve state-of-the-art results, making it a valuable tool for applications such as drone-based glass curtain wall crack detection, where both performance and efficiency are important.

## 5. Conclusions

This paper introduces GlassCurtainCrackDeblurNet, a novel GAN-based and enhanced U-shaped Transformer method specifically designed for the deblurring of drone images capturing glass curtain wall cracks. Through rigorous experimental comparisons, our proposed method demonstrated a superior performance over other widely used deblurring techniques on the GoPro Dataset.

To further optimize the deblurring performance of our method for drone imagery of glass curtain cracks, we specifically established the GlassCurtainCrackDeblur Dataset. Following the training of our method and other established deblurring methods on this dataset, our method once again achieved the best qualitative and quantitative results, with a notably reduced computational time compared to its competitors.

We believe that the combination of our proposed GlassCurtainCrackDeblurNet and the GlassCurtainCrackDeblur Dataset will enable more effective removal of blur caused by drone flight, leading to more accurate crack detection in glass curtain walls. This advancement holds promise for enhancing the safety and reliability of high-altitude inspections, particularly in urban environments where the number of high-rise buildings continues to grow.

## Figures and Tables

**Table 1:** Key characteristics in the GlassCurtainCrackDeblur Dataset.

Key Characteristics	Number
Without cracks	2899
Simple cracks	828
Complex cracks	414
single-directional blur	3106
multi-directional blur	1035

**Table 2:** The quantitative evaluation on the GoPro Dataset.

Method	MAE	PSNR (dB)	SSIM	Time (s)
DeblurGANv2	0.0401	25.51	0.80	0.12
Method [35]	0.0384	27.85	0.85	1.46
Restormer	0.0215	28.94	0.88	1.97
GlassCurtainCrackDeblurNet (ours)	0.0198	29.76	0.89	0.93

**Table 3:** The quantitative evaluation on the GlassCurtainCrackDeblur Dataset.

Method	MAE	PSNR (dB)	SSIM	Time (s)
DeblurGANv2	0.0521	25.36	0.81	0.71
Method [35]	0.0352	27.97	0.84	1.49
Restormer	0.0207	28.66	0.87	18.32
GlassCurtainCrackDeblurNet (ours)	0.0161	30.40	0.91	0.92

**Table 4:** The comparison between the purposed method with other approaches.

Method	Image Refinement	Structural Awareness	Instant Inference Ability
DeblurGANv2			√
Method [35]	√		
Restormer		√	
GlassCurtainCrackDeblurNet (ours)	√	√	√

“√” means the model owns this ability.

## Data Availability

The raw data supporting the conclusions of this article will be made available by the authors on request.
